# *Fusarium oxysporum* f. sp. *niveum* Molecular Diagnostics Past, Present and Future

**DOI:** 10.3390/ijms22189735

**Published:** 2021-09-08

**Authors:** Owen Hudson, James C. Fulton, Alexi K. Dong, Nicholas S. Dufault, Md Emran Ali

**Affiliations:** 1 Department of Plant Pathology, University of Georgia, Tifton, GA 31793, USA; owen.hudson@uga.edu (O.H.); Alexi.Dong@uga.edu (A.K.D.); 2 Department of Plant Pathology, University of Florida, Gainesville, FL 32611, USA; nsdufault@ufl.edu

**Keywords:** fusarium wilt of watermelon, *Fusarium oxysporum* f. sp. *niveum*, comparative genomics, diagnostics, distribution, race differentiation, effector profile

## Abstract

Watermelon is an important commercial crop in the Southeastern United States and around the world. However, production is significantly limited by biotic factors including fusarium wilt caused by the hemibiotrophic fungus *Fusarium oxysporum* forma specialis *niveum* (Fon). Unfortunately, this disease has increased significantly in its presence over the last several decades as races have emerged which can overcome the available commercial resistance. Management strategies include rotation, improved crop resistance, and chemical control, but early and accurate diagnostics are required for appropriate management. Accurate diagnostics require molecular and genomic strategies due to the near identical genomic sequences of the various races. Bioassays exist for evaluating both the pathogenicity and virulence of an isolate but are limited by the time and resources required. Molecular strategies are still imperfect but greatly reduce the time to complete the diagnosis. This article presents the current state of the research surrounding races, both how races have been detected and diagnosed in the past and future prospects for improving the system of differentiation. Additionally, the available Fon genomes were analyzed using a strategy previously described in separate formae speciales avirulence gene association studies in *Fusarium oxysporum* races.

## 1. Introduction

As a single species, *Fusarium oxysporum* is rated the fifth most important fungal plant pathogen in the world [1]. Within the *Fusarium oxysporum* species complex (FOSC), there are over 106 known formae speciales which infect more than 100 different hosts causing vascular wilts [1,2]. They are soilborne, can survive for long periods, are often unaffected by chemical management, and can evolve to overcome host resistance quickly [3,4]. As hemibiotrophic pathogens, they not only cause yield loss, but also result in total plant death and crop loss [5]. Recent research trends in several formae speciales of the FOSC have focused on the molecular analysis of pathogenicity genes, whole genome sequence analysis, and proteomics, to understand the infection process. It is the case that FOSC isolates are ubiquitous in the soil around the world, but are largely nonpathogenic [6,7]. The mobility and plasticity of the FOSC genomes is also of particular interest to determine if nonpathogenic strains can gain pathogenic function either by traditional mutation or through chromosomal movement and exchange [8,9].

**Figure 1:**
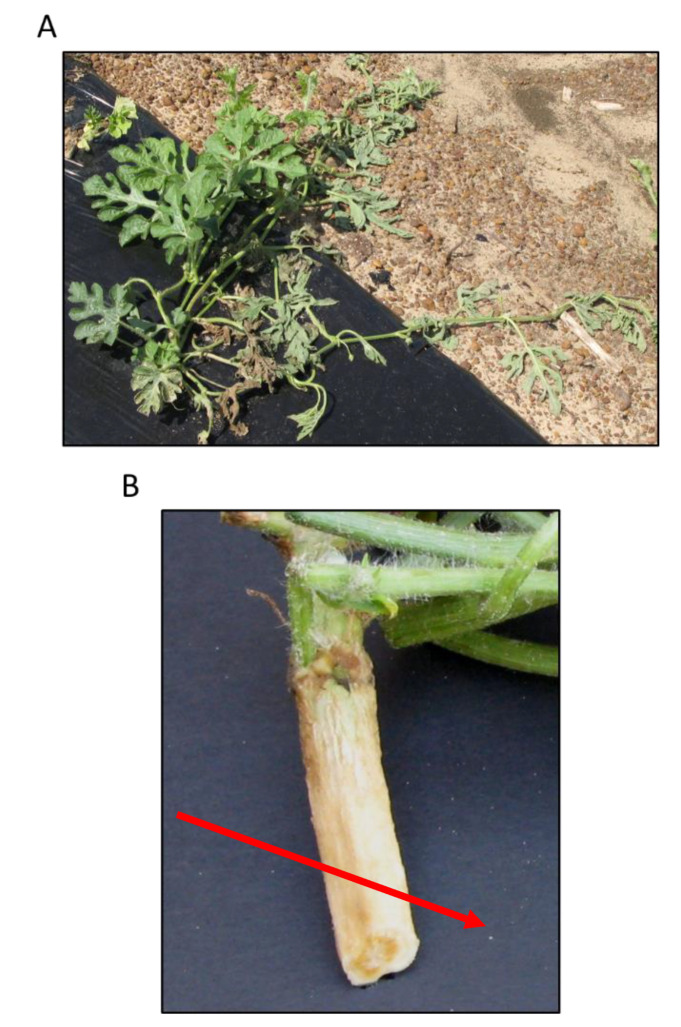
Fusarium wilt symptoms (**A**) in the field, and (**B**) in the vasculature (arrow).

In the Southeastern United States, *Fusarium oxysporum* forma specialis *niveum* (Fon) is widely distributed and causes major yield losses in watermelons, the only host of Fon [10,11]. There are additional FOSC formae speciales that infect watermelon but also infect other crops [12]. Fusarium wilt of watermelon, the disease caused by Fon, results in vascular clogging leading to wilting in one or two infected runners (Figure 1). Over time, the infection intensifies leading to total plant wilting and eventual necrosis [13]. The cool and moist springtime climate (below 70 °F) common to the Southeastern US is favorable for disease spread and initiation and occurs during the watermelon seedling stage. Thus, Fon is responsible for significant damping off in seedlings, and outbreaks primarily occur early (February–April) in the season [14].

**Figure 2:**
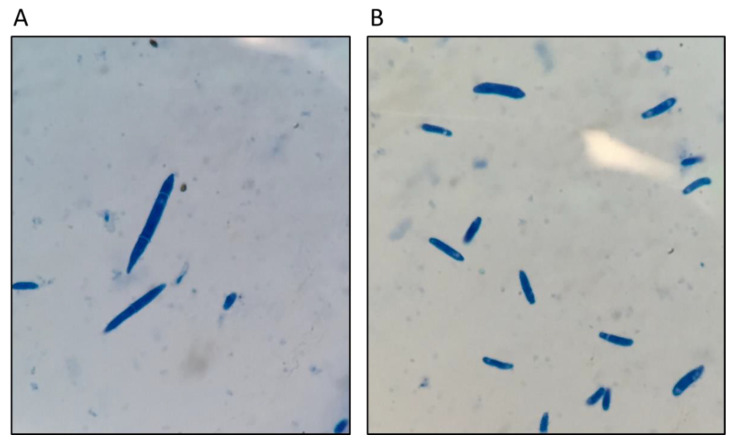
Microscopic morphology of Fon (**A**) macroconidia, and (**B**) microconidia.

Three main spore types exist for *Fusarium oxysporum* f. sp. *niveum*: microconidia, macroconidia (Figure 2), and chlamydospores. Chlamydospores are the primary infective propagule that survive from one season to the next. They arise from both hyphae and from groups of macroconidia forming sporodochia. Chlamydospores are formed in times of stress when nutrients for survival run low and germinate when they detect an increase of nutrients from plants or seeds. Not only do chlamydospores often provide the initial infection on the watermelon host, but can also survive for up to a decade or longer in the soil [15,16,17]. Once a chlamydospore detects plant exudates, the spore germinates. The root exterior is then colonized by the chlamydospore hyphae which occurs on both host and nonhost plants.

When on a susceptible host plant, the mycelia then penetrate the root to the xylem where the infection continues and other spore types are produced. Penetration of the plant host can occur through natural openings/cracks in the root but is increased by physical wounds that occur in the root surface. Microconidia are generated primarily in the xylem space and travel with the flow of water from one vessel cell to the next, germinating at the end and producing more microconidia on the opposite side of the vessel cell wall. Cell wall-degrading enzymes are produced and act as clogs for water transport. Many types of enzymes have been associated with the initiation of the disease process of many Fusarium wilt pathogens and good summaries of these can be found in Martyn, 2014 [13]. These enzymes, along with the plant host defense of limiting the spread of the microconidia by the building up of tyloses, cause the wilting symptoms typical of Fon infections in the field. Decreased water transport and vascular necrosis from the systemic spread of the pathogen cause symptomatology that is used for diagnosis. In susceptible plants, the pathogen will eventually cause total necrosis and death of the plant. Tyloses produced by the plants can play an important role in the successful defense reactions of the plant against the pathogen by limiting its spread [18]. 

Some formae speciales are further divided into races (usually) to indicate cultivar-specific pathogenicity, however, this terminology is not universally accepted and thus raises questions about appropriate nomenclature [2]. Subdivisions of race within an individual forma specialis are defined by their pathogenicity on specific and newly susceptible cultivars. Races are characterized by their ability to overcome a specific cultivar’s host resistance [2,19,20]. This reaction also can describe virulence, as how the differential interactions linked to virulence genes (R-genes) govern the susceptibility of the previously resistant cultivars. Aggressiveness is a quantitative component of pathogenicity and does not have a connection to race differentiation [21]. Resistant watermelon cultivars have been developed, but new resistant races of Fon have arisen [22]. Fon has four recognized races: 0, 1, 2, and 3. Each subsequent race is determined by its ability to cause infection on previously resistant cultivars, with race 3 having the largest range of pathogenicity. Although the specific avirulence gene responsible for overcoming cultivar resistance is not known in most races, the ability to cause infection should be characterized as virulence rather than pathogenicity as their differentiation rests on a 1–9 scale of disease rating. The aggressiveness of Fon has been thought to positively correlate with increased cultivar range, meaning race 3 isolates both have a larger range of pathogenicity (virulence) and aggressiveness than race 1 isolates. This has been argued due to multiple isolates of the same race being tested and showing variation within their aggressiveness [23,24].

**Figure 3:**
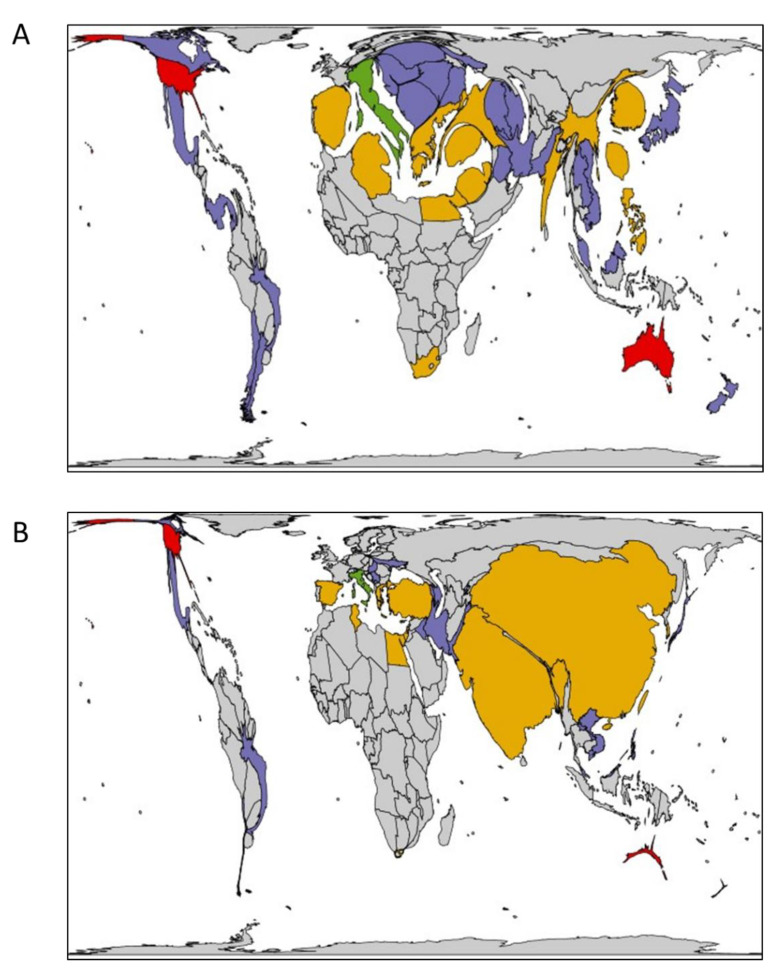
Global Fon distribution. Countries are colored based on highest race detected: green, race 1; yellow, race 2; red, race 3; and purple, race not reported. (**A**) Country area proportional to watermelon yield (hg/hectare), and (**B**) country area proportional to watermelon production (metric tons). Images produced using an online program which scales images depending on a determined variable while maintaining the original object boundaries as computed by a flow-based algorithm [27].

Fon is found in most, if not all, watermelon growing regions in the world [25]. It is present in the six possible continents and is recorded in 44 countries (Figure 3). The majority of countries that report the presence of Fon do not specify the race (only 13 nations report specific races) which could suggest the presence of additional races or levels of virulence of detected isolates. Distinct countries use different watermelon cultivars for race differentiation purposes as well, complicating the comparison across continents and countries [26]. This goal of this review is to discuss the available diagnostic and race-differentiation methods for Fon while also providing a brief overview of presence, distribution, and management.

## 2. Diagnostics

### 2.1. Bioassays

Forma specialis status of Fon was designated based on the specificity of Fon to be solely pathogenic on watermelon and not infecting closely related cucurbit hosts [11]. Race differentiation is also done with a bioassay, but using multiple different cultivars which have a corresponding reaction to each particular race of the pathogen (Table 1) [28].

**Figure 4:**
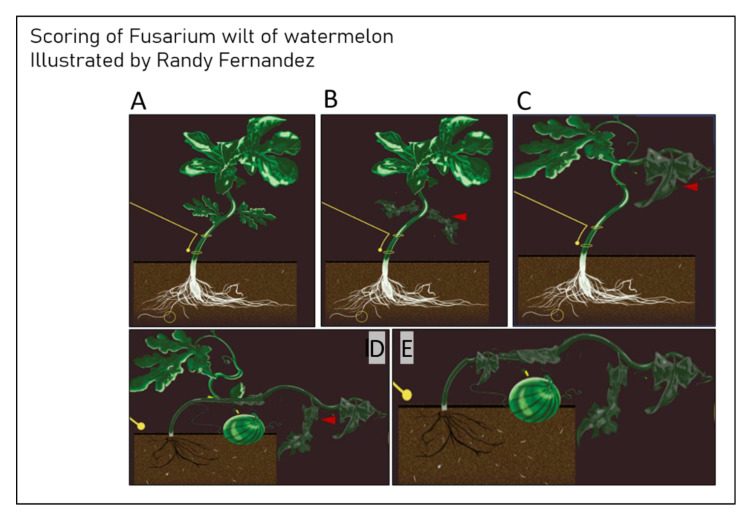
Visual differences in Fon symptoms on watermelon plants for scoring disease. (**A**) Score of 0, asymptomatic plants. (**B**) Score of 3, plants have cotyledon lesions. (**C**) Score of 5, plants are slightly wilted and stunted. (**D**) Score of 7, plants are severely wilted and stunted. (**E**) Score of 9, plants are dead.

Susceptibility is determined based on a disease rating scale from 1–9, 0 for asymptomatic plants, 3 for plants with cotyledon lesions, 5 for plants with slight wilting and stunting, 7 for plants with severe wilting and stunting and 9 for dead plants. Plants rated as 0 are classified as resistant, 1 or 3 as intermediate resistant and 5, 7 or 9 as susceptible [10]. Issues arise when assigning a value to symptoms that may overlap or skip, for example, a plant wilting with no lesions, or lesions and necrosis with no stunting. Some isolates are characterized as nonpathogenic but yet still Fon. Additional problems exist when assigning a race based on the virulence that determines the level of pathogenicity; for example, an isolate which is highly virulent on Sugar Baby, mildly virulent on Charleston Grey and Calhoun Grey, and highly virulent on PI-296341-FR. While this may result in a race 3 assignment, there are clearly other mechanisms that contribute to the disease reaction and host response that are still unknown. Other variable factors that contribute to the consistency of the bioassay include substrate type, temperature, humidity, propagule concentration, age of the plant, water usage, and correct cultivar (Figure 4). Each one of these factors can change the outcome of a single reaction so a significant number of replicates are needed. That said, the replicability of these assays remains difficult, partially for labs that may not have the resources to adapt to the required factors, and partially due to the bioassay description in the literature failing to mention the details of all the aforementioned aspects. Because of the possible variations in both the bioassay inputs and results, molecular methods to detect and differentiate have been a recent focus. The design of molecular markers that remain consistent over time and unchanged between geographically distinct isolates is key for a successful Fon diagnosis. Alternatively, markers amplifying differences using only local isolates can be misinterpreted when used outside of that region. Because race differentiation is done on the basis of pathogenicity and virulence, when designing markers for race differentiation, one must additionally link those markers to specific genes involved in the pathogenicity.

### 2.2. Molecular Assays

Fon-specific molecular detection methods targeted the commonly used fungal barcoding region, the internal transcribed spacer (ITS). This work was done by Zhang et al. (2005), and the PCR primer set was labeled Fn-1/Fn-2 [32]. While this marker greatly improves the diagnostic ability of researchers working on Fon, it has been discussed whether the published marker may amplify more recent FOSC members in addition to Fon [33] (personal communication). A second PCR marker was developed by Lin et al. (2010) from a DNA sequence generated by random amplification of polymorphic DNA (RAPD) that could differentiate Fon from the other forma speciales of FOSC. They additionally showed that Fn-1/Fn-2 amplified nontarget FOSC members from Taiwan, but that the new marker, Fon-1/Fon-2, would not. Overall, it is useful to use both markers when resources are available, but the more recent marker, that of Lin et al., appears to be more specific. Keinath et al. (2020), reported that nonpathogenic Fon isolates may not be detected by the Fon-1/Fon-2 primer set [26]. It should also be noted that in watermelon growing regions, the traditional detection of Fon is more often done in the field and the above described markers do not produce amplicons in the presence of DNA from other pathogens causing similar symptoms in watermelons [4,34].

An additional Fon-specific marker for TaqMan real-time PCR has been developed by van Dam et al. (2018), which targets a region of the elongation factor 1-alpha (EF1α) [35]. While requiring additional materials and instruments to process, the assay showed no cross-reaction with nontarget strains which included a large number of FOSC members. Groups of formae speciales were also grouped based on candidate effector genes and were found to correlate with host specificity, a possible strategy for race differentiation that is discussed later.

More difficult and subsequently more important for growers and breeders, is Fon race differentiation. While the traditional bioassay method has been discussed, there exist a few molecular markers that claim the ability to differentiate between several of the races present in the literature. The first, by Niu et al. (2016), identified the avirulence gene Secreted in Xylem 6 (*SIX6*) in races 0 and 1, but was absent in race 2 [36]. Race 3 was not tested in their study, but in subsequent research, race 3 was found to contain *SIX6* [24]. Mutation studies with this gene increased virulence in race 1 isolates without *SIX6* and reduced virulence in race 2 isolates when given *SIX6*, drawing a connection between isolate virulence and the Secreted in Xylem gene family which is discussed further below. The absence of *SIX6* as detected by *FONSIX6* specific markers has been used for race 2 differentiation, with races 0, 1 and 3 showing a positive reaction. In the literature, isolates with levels of pathogenicity that would normally identify the isolate as race 2 seldom use the marker for *SIX6* to determine a correlation between the bioassay and the marker. When carried out, it does not appear to correlate at a high percentage [26], which suggests multiple genetic contributions for pathogenicity, of which *SIX6* may be one.

**Figure 5:**
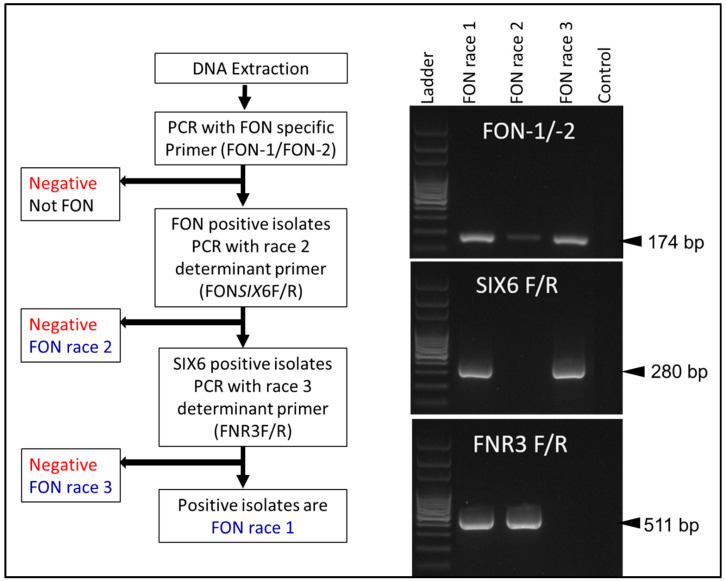
Molecular method of Fon race differentiation adopted from Hudson et al. (2021) [24].

Further race differentiation research focused on race 3 differentiation similar to that of race 2. Hudson et al. (2021) [24] sequenced the whole genomes of suspected race 1, 2 and 3 isolates before determining the regions that could be used for differentiation. The primer set FNR3F/R, while targeting a chromosomal region involved in pathogenicity, did not directly link the amplified region to any gene function other than coding for a hypothesized protein (Figure 5). FNR3F/R does not provide race 0 identification, nor identification of nonpathogenic Fon strains. FNR3F/R amplifies a region in race 1 and race 2 isolates but does not amplify anything in race 3 isolates. Using Fon-1/Fon-2 and FONSIX6F/R primer sets alongside FNR3F/R, races 1, 2 and 3 can be differentiated. Based on a sampling of over 90 race typed Fon isolates, 89% of race 1 isolates were predicted by the marker, 80% of race 2 isolates, and only 60% with the race 3 marker. Problems with this marker include the correlation between the bioassay results and the molecular results, as well as not including nonpathogenic or race 0 isolates. In order to determine the function of the gene targeted with FNR3F/R, knockout mutants lacking the genomic region must be made for various isolates [36,37,38].

### 2.3. Evaluation of Diagnostic Methods

Overall, *Fusarium oxysporum* species identification and diagnosis is improving in accuracy and speed as the available genetic resources increase. Diagnosis to the forma specialis level is clearly improved using molecular tools as there are multiple options which can selectively amplify DNA from Fon isolates instead of DNA from genetically similar formae speciales or other symptomatically similar pathogens [32,35]. While DNA extraction and PCR assays require specialized tools, they have become more routine for diagnosis in plant pathology and more widely distributed [39,40,41]. In comparison, the bioassay requires more resources and time to grow multiple different crop hosts before completing the diagnosis (Table 2 and Table 3) [23,24,42]. Even then, nonpathogenic strains may not be isolated. It is less clear which method provides more accurate and rapid differentiation of Fon races. This is particularly clear when the studies comparing the two methods do not have matching results to a significant degree. The complexity of unstable nomenclature between virulence, pathogenicity, and aggressiveness, contribute to complicating interpretation of the bioassay, particularly of novel results. The lack of whole genome sequencing decreases the confidence that one has when using molecular assays and genes encoding hypothetical proteins used for differentiation must be confirmed to have direct involvement in the infection process. Resistance to various *Fusarium* species is identified as quantitative in multiple hosts [43,44,45,46]. Thus, amplification of a single gene or region is unlikely to fully capture the pathogenicity of the isolate. It is reasonable to say that no currently available method can rapidly and accurately differentiate Fon races, but rather the previously designed markers likely amplify the regions that contribute to pathogenicity and therefore may be of assistance to Fon diagnosis by identifying pathogenicity-related genes [24,36]. Several proteins involved in disease progression have been identified in Fon and *Fusarium oxysporum* (Fo) more generally, namely the Secreted-in-Xylem proteins. Certainly, some of these genes contribute to pathogenicity, but for differentiation purposes, multiple genes must be identified as unique contributors to the infection. This can alternatively be achieved by determining the SIX gene profile: what set of SIX genes is present in each race and if a unique combination is found, how it relates to virulence.

### 2.4. Genomics Diagnostics

SIX [47,48,49] proteins have been shown to be strongly correlated with disease progression and virulence in hosts infected by Fo species [50]. The exact mechanism and how they function to cause disease is unknown, however, research has highlighted their possible involvement in interfering with host signal transduction and the Jasmonic acid-mediated response to detecting the presence of PAMPS [51,52]. SIX proteins are characterized by containing fewer than 300 amino acids, an abundance of cysteine residue, and the inclusion of a secretion peptide signal. The exact composition of SIXs and their homologs can predict the pathogen host range including formae speciales and races and have been used to develop molecular diagnostic assays [53,54,55,56]. Since the first suggested use of distinguishing races and formae speciales by its application as proposed by Lievens et al., research in FOL showed that the three described races were differentiated by the presence/absence of SIX1, 3, and 4 genes [57,58]. In another example, Czislowski et al. who showed that the SIX absence/presence profile was strongly correlated with the pathogenicity of known Foc lineages [59]. Similarly, it is hypothesized that the virulence demonstrated by various Fon isolates on differential cultivars is likely the result of specific permutations of SIX, or other, effectors. For example, Table 4 shows the distribution and identity of known SIX effectors in all publicly available Fon genomes (SAMN15791673, SAMN15791674, and SAMN15791675). While the race classification for some of these assemblies is known, most are not. BLAST searches reveal seven unique combinations which suggests either additional races or effectors with a negligible contribution to pathogenicity. Clearly, additional isolate genomes with race classification information is necessary for the application of this method to sufficiently differentiate the genetic basis underlying the variability of virulence in Fon. 

Whole genome sequencing studies have also indicated the importance of entire chromosomes. For example, Ma et al. showed that chromosomes, 3, 6, 14, and 15 were uniquely distinct from the rest of the genome by the presence of many transposable elements and demonstrated the ability to confer pathogenicity in previously avirulent strains after the transformation of chromosome 14 from strain *Fol*4287 [60]. Coincidentally, the aforementioned SIX genes are often located on these chromosomes and can be predicted, based on the presence of specific miniature inverted-repeat transposable elements (MITE) called miniature impalas, or transposons belonging to the *Tc1/mariner* superfamily of class II transposons [61,62,63]. Building on this advance, van Dam et al. (2016) identified genes encoding hypothesized effectors in whole genome sequences of five melon-infecting formae speciales, based on their proximity to transposable elements, the presence of a secretion signal, and modest size, to develop effector profiles unique to each forma specialis [56]. Through the analysis of these unique effector profiles, van Dam et al. (2018) were able to develop PCR primers that distinguished between seven cucurbit-infecting formae speciales [35]. Recently, whole genome sequencing has allowed for the identification of two lineages based on effector profiles for *Fusarium oxysporum* f. sp. *spinaciae* causing wilt in spinach [64]. Using a whole genome comparison approach, Batson et al., identified three distinct populations (nonpathogenic, and two races) of *Fusarium oxysporum* f. sp. *spinaciae* isolates. Similar to the van Dam approach, they first identified genes encoding putative effectors from 14 sequenced genomes from both nonpathogenic and pathogenic isolates. Based on the absence/presence of characteristics of these genes coding for these effectors in the profiles three unique lineages were described during phylogenetic analysis.

This investigation into methods of race differentiation and specific pathogen detection has far reaching implications in plant pathology. As the importance of speed and accuracy continues to grow in diagnostics, having well-rooted and easy-to-follow methodologies behind the diagnostics also increase in importance. Effector profiles, as suggested in this review, provide a clear process that is easily understandable to multiple levels of researchers about how a diagnostic method is designed. Additionally, plant breeders that are not so well versed in molecular diagnostics can adapt the pathogenicity-related components into different breeding programs that now have one or more target(s) for increased accuracy. Researchers working on other *Fusarium* species can also gain by using the methods of differentiation described here or by avoiding some of the problems that have arisen in this pathosystem.

## 3. Conclusions

Based on the limited host range of Fon, it likely has a reduced, yet specific, set of effectors that allow for setting up a disease to watermelon. The set of effectors present in Fon which permit infection in watermelons is likely shared with other *Fusarium* species whose host range includes watermelon (*F.o. melonis*, *F.o. cucumerinum*, possibly even *F. solani cucurbitae*). However, the exact, unique complement of effectors which overcome cultivar-specific host resistance remains uncharacterized. Because of this perspective, we suggest that, moving forward, an effort should be made to increase the number of available Fon genomes with or without race ID. Once a significant number of genomes are available from variable geographic locations, the profiling done here should be repeated with the new isolates to determine the number of distinct gene profiles. Effector function on Fon virulence affecting available watermelon cultivars should then be examined and each SIX gene’s function on virulence can be determined using the susceptible cultivars. Connections then can be made for breeders based on corresponding resistance (R) genes in watermelon and race designation can be done using these genetic interactions.

While it is true that the designation of specific races based on an effector gene profile requires more steps and greater computational resources, once determined, the most important genetic combinations can be adapted for molecular diagnostic methods [35]. Additionally, the ability for FOSC members to exchange chromosomal elements and perform horizontal gene transfer no longer interferes with molecular diagnostics because detection will be based on the specific genes which govern virulence, not simply conserved regions with other metabolic functions. Effector genes that are common to all isolates can then be analyzed compared to the other *Fusarium* species that infect watermelon to design new and improved molecular markers for Fon-specific detection.

It has been noted by several researchers studying Fon that the difference between races 0 and 1 may be quantitative rather than qualitative, but that race 2 is distinct [25]. These perspectives were written before the appearance of race 3, but it would still appear that multiple genes facilitate the virulence of any given race and that a particular combination of those virulence genes may dictate the ability of a Fon isolate to cause infection. A single identifiable gene required for infection on a resistant watermelon cultivar is needed to consider the resistance qualitative; *FONSIX6* has been studied as that target gene to isolate race 2 Fon isolates [36]. Confirmation of *FONSIX6* as the only gene responsible for infection on the resistant cultivars (i.e., qualitative disease resistance) must occur by demonstrating that the presence or absence of *FONSIX6* causes no difference in aggressiveness against susceptible cultivars, but only allows for the infection to take place on the previously resistant cultivars. If, when *FONSIX6* is absent, the Fon isolate is shown to increase the aggressiveness on previously susceptible cultivars and that it overcomes resistance, then the resistance is quantitative and breeders must incorporate resistance against all avirulence genes involved in the disease process.

## Figures and Tables

**Table 1:** Common cultivars used for race differentiation. R = resistant and S = susceptible. Cultivars are repeated when contradictory claims occur and are marked with an asterix *.

	Race 0	Race 1	Race 2	Race 3	Reference
Sugar Baby	S	S	S	S	[1,3,4]
Black Diamond	S	S	S	S	[1,3,5]
Charleston Gray	R	S	S	S	[1,5]
Crimson Sweet	R	S	S	S	[1,5]
Mickey Lee	R	R	S	S	[4]
Dixielee *	R	S	S	S	[2]
Dixielee *	R	R	S	S	[3]
Allsweet *	R	S	S	S	[2]
Allsweet *	R	R	S	S	[1,3,5]
Calhoun Gray	R	R	S	S	[1,3,5]
PI-296341-FR	R	R	R	S	[1]

Note: Charleston gray and Crimson sweet were not effective because of overlapping percentage of infection—3. References: [11,29,30,31].

**Table 2:** Primers used for molecular detection and differentiation of Fon isolates.

Purpose of Assay	Primers	Sequence (5′-3′)	Product Size (bp)	Source
Fon-specific primer	Fon-1	CGATTAGCGAAGACATTCACAAGACT	174	Lin et al., 2010 [33]
	Fon-2	ACGGTCAAGAAGATGCAGGGTAAAGGT		
Race 2 differentiating primer	FONSIX6F	CGCTCTTATCGCATCAATCT	453	Niu et al., 2016 [36]
	FONSIX6R	GGGTTGACTGAGGTCGTGGT		
Race 3 differentiating primer	FNR3F	CGGCTTTCCTCTGTCAGATAGT	511	Hudson et al., 2021 [24]
	FNR3R	TAGTGAGGTCCATGCCACGAA		

**Table 3:** Comparison between diagnostic techniques available for Fusarium wilt of watermelon.

Diagnostic Technique	Advantages	Disadvantages
Field observation	Fastest, low training needs	Not accurate, no distinct race determination
Culturing and microscopy	Only basic lab instruments required. *Fusarium* sp. confirmation rapidly.	Skills for culturing, isolation, and microscopy required. Knowledge of morphology required. No species or race level determination possible
Greenhouse bioassay	Race identification possible. Highly controlled environment allowing for statistical analysis. Current standard for evaluation of Fon isolates	Culturing of Fon required. Specific cultivar’s seeds, controlled greenhouse environment, sterile locations required. Multiple replications needed for confirmation. Several months for final results
Molecular (PCR based)	Ability to rapidly determine species and often race level of Fon. Very fast to determine Fon races.	Access to molecular lab and molecular lab equipment. Training in all PCR methods required. Not confirmed to be able to distinguish all races. Unable to determine race if multiple genes involved.
Gene profile	Able to determine all possible variations of effectors present and active in a Fon isolate. Theoretically exact race determination possible even if resistance is quantitative. Once determined, the ability can be converted to conventional molecular methods (PCR).	Multiple gene sequencing of individual isolates required. Higher level of knowledge for sequencing analysis and multigene sequencing required. Instruments additionally more complex and expensive. More annotations and whole genome sequencing required.

**Table 4:** Secreted-in-xylem (SIX) effector profile for *Fusarium oxysporum* f.sp. *niveum* (Fon) isolates taken from whole genome sequences.

Putative Race	0	1		2	3		Unknown								
Isolate Name	110407_3_1_1	150523	R1	R2	150524	R3	Fon002	Fon005	Fon010	Fon013	Fon015	Fon019	Fon020	Fon021	Fon037
Accession number	GCA_01959 3455.1 [65]	GCA_01959 3445.1 [65]	GCA_0146 02815.1 [66]	GCA_0146 02775.1 [66]	GCA_0195 93505.1 [65]	GCA_0146 02795.1 [66]	GCA_0017 02745.1 [9]	GCA_0017 02505.1 [9]	GCA_0017 02785.1 [9]	GCA_0017 02775.1 [9]	GCA_0017 02795.1 [9]	GCA_0017 02715.1 [9]	GCA_0017 02805.1 [9]	GCA_0017 02865.1 [9]	GCA_0017 02845.1 [9]
SIX1	Absent	Absent	Absent	Absent	Absent	Absent	Absent	Absent	Absent	Absent	Absent	Absent	Absent	Absent	Absent
SIX2	Absent	Absent	Absent	Absent	Absent	Absent	Absent	Absent	Absent	Absent	Absent	Absent	Absent	Absent	Absent
SIX3	Absent	Absent	Absent	Absent	Absent	Absent	Absent	Absent	Absent	Absent	Absent	Absent	Absent	Absent	Absent
SIX4	Absent	Absent	Absent	Absent	Absent	Absent	Absent	Absent	Absent	Absent	Absent	Absent	Absent	Absent	Absent
SIX5	Absent	Absent	Absent	Absent	Absent	Absent	Absent	Absent	Absent	Absent	Absent	Absent	Absent	Absent	Absent
SIX6	Absent	Present	Present	Absent	Absent	Present	Absent	Present	Absent	Present	Absent	Absent	Present	Absent	Absent
SIX7	Absent	Absent	Absent	Absent	Absent	Absent	Absent	Absent	Absent	Absent	Absent	Absent	Absent	Absent	Absent
SIX8	Absent	Present	Present	Present	Present	Present	Absent	Present	Present	Present	Absent	Absent	Present	Present	Absent
SIX9	Present	Present	Present	Present	Present	Present	Absent	Present	Present	Present	Present	Present	Present	Present	Absent
SIX10	Absent	Absent	Absent	Absent	Absent	Absent	Absent	Absent	Absent	Absent	Absent	Absent	Absent	Absent	Absent
SIX11	Absent	Present	Present	Present	Present	Present	Present	Present	Present	Present	Present	Present	Present	Present	Present
SIX12	Absent	Absent	Absent	Absent	Absent	Absent	Absent	Absent	Absent	Absent	Absent	Absent	Absent	Absent	Absent
SIX13	Absent	Present	Present	Absent	Absent	Present	Present	Present	Present	Present	Present	Present	Present	Present	Present
SIX14	Absent	Absent	Absent	Absent	Absent	Absent	Present	Absent	Absent	Absent	Absent	Absent	Absent	Absent	Absent

## Data Availability

Data sharing is not applicable to this article.

## Abbreviations

Fon	*Fusarium oxysporum* f. sp. *niveum*
SIX	Secreted in Xylem
FOSC	*Fusarium oxysporum* species complex
PCR	Polymerase chain reaction
RAPD	Random amplification of polymorphic DNA
PAMP	Pathogen associated molecular patterns
FOL	*Fusarium oxysporum* f. sp. *lycopersici*
BLAST	Basic local alignment search tool
MITE	Miniature Inverted-repeat Transposable Elements
